# An Investigation towards Coupling Molecular Dynamics with Computational Fluid Dynamics for Modelling Polymer Pyrolysis

**DOI:** 10.3390/molecules27010292

**Published:** 2022-01-04

**Authors:** Timothy Bo Yuan Chen, Ivan Miguel De Cachinho Cordeiro, Anthony Chun Yin Yuen, Wei Yang, Qing Nian Chan, Jin Zhang, Sherman C. P. Cheung, Guan Heng Yeoh

**Affiliations:** 1 School of Mechanical and Manufacturing Engineering, University of New South Wales, Sydney, NSW 2052, Australia; timothy.chen@unsw.edu.au (T.B.Y.C.); i.decachinhocordeiro@unsw.edu.au (I.M.D.C.C.); qing.chan@unsw.edu.au (Q.N.C.); jin.zhang6@unsw.edu.au (J.Z.); g.yeoh@unsw.edu.au (G.H.Y.); 2 School of Energy, Materials and Chemical Engineering, Hefei University, 99 Jinxiu Avenue, Hefei 230601, China; yangwei@hfuu.edu.cn; 3 School of Aerospace, Mechanical and Manufacturing Engineering, RMIT University, Melbourne, VIC 3083, Australia; chipok.cheung@rmit.edu.au; 4 Australian Nuclear Science and Technology Organization (ANSTO), Locked Bag 2001, Kirrawee DC, NSW 2232, Australia

**Keywords:** combustion, computational fluid dynamics, detailed chemistry, flame retardants, molecular dynamics, pyrolysis

## Abstract

Building polymers implemented into building panels and exterior façades have been determined as the major contributor to severe fire incidents, including the 2017 Grenfell Tower fire incident. To gain a deeper understanding of the pyrolysis process of these polymer composites, this work proposes a multi-scale modelling framework comprising of applying the kinetics parameters and detailed pyrolysis gas volatiles (parent combustion fuel and key precursor species) extracted from Molecular Dynamics models to a macro-scale Computational Fluid Dynamics fire model. The modelling framework was tested for pure and flame-retardant polyethylene systems. Based on the modelling results, the chemical distribution of the fully decomposed chemical compounds was realised for the selected polymers. Subsequently, the identified gas volatiles from solid to gas phases were applied as the parent fuel in the detailed chemical kinetics combustion model for enhanced predictions of toxic gas, charring, and smoke particulate predictions. The results demonstrate the potential application of the developed model in the simulation of different polymer materials without substantial prior knowledge of the thermal degradation properties from costly experiments.

## 1. Introduction

Fire risks associated with lightweight building materials have continuously threatened building occupants, the environment, and properties [1,2]. The rise in material complexity has also generated new challenges and requirements concerning fire safety protection systems. Subsequently, it has driven significant interest in developing robust numerical tools to effectively assess the fire behaviours and performance of these combustible materials in fire investigation studies and establish safe use guidelines, especially for the rapid development of flame retardants, prediction of toxicity emissions and self-extinguishing behaviours.

The application of Computational Fluid Dynamic (CFD) modelling on building fires has gained massive adoption due to the rapid advancement of computational power and offers a cost-effective method to analyse material fire performance compared to conventional fire testing [3,4,5,6,7]. Specifically. Large-eddy simulation (LES)-based fire field modelling has become dominant in numerical studies of fire dynamics, with generalised fire codes, such as Fire Dynamics Simulator (FDS) [8], FireFOAM [9] and several others [10,11]. Typically, thermogravimetric analysis (TGA) is applied to extract the decomposition kinetics of polymers [12], while the flammability and ignitability of the material can be studied via Cone Calorimetry [3]. Through analysing the thermal degradation at multiple heating conditions (i.e., constant heating rates) [13,14], the resultant pyrolysis rate can be expressed in the form of Arrhenius expression, similar to those applied for gas-phase reactions. For other structural properties, there are Scanning Electron Microscopy and Transmission Electron Microscopy, X-ray Photoelectron Spectroscopy and Small-angle Neutron Scattering [15,16,17]. There are many successful CFD studies on the fire behaviour of polymer materials. Nguyen et al. [18] investigated the fire resistance of glass fibre reinforced polymer composite via a methodology incorporating experimental (TGA, cone calorimetry and single item burn test) and numerical simulation. The experimental results were used as input parameters and validation to construct the numerical model. The model then provided more detailed insight into the burning process and flame spread behaviour. Dutta et al. [19] conducted a numerical study on natural fibre composites. The pyrolysis kinetics were extracted from TGA experiments and applied in an FDS model of the Cone Calorimeter under horizontal and vertical orientations. More recently, Yuen et al. [4,20] coupled an in-house pyrolysis model with kinetics data extracted via a genetic algorithm to study the thermal decomposition of flame-retardant polyurethane foams. The authors highlighted that a more detailed pyrolysis breakdown of gas products from thermal decomposition could improve the accuracy of smoke and Carbon Monoxide predictions. CFD modelling of polymer pyrolysis is also extensively applied in hybrid rocket engines [21]. Tyurenkova et al. [22,23,24,25] conducted a comprehensive series of numerical studies on solid fuel pyrolysis in hybrid rocket engines and investigated the regression behaviour in different flow regimes. It has been found that turbulent transfer coefficients, such as Prandtl number play an essential role to model the gas-solid surface interactions and the regression rate.

Although CFD techniques are widely used to simulate the burning of building materials, there are still many limiting factors from current modelling frameworks. For instance, obtaining material input parameters is one of the major difficulties in conducting fire modelling. It requires quality and reliable data obtained through multiple fire experiments, which are costly and destructive. Furthermore, there are limited techniques to extract the actual chemical composition of emitted volatiles to properly characterise the combustion chemistry in the gas phase and model the solid pyrolysis process [26]. Consequently, single-step gas-phase combustion reactions are often applied instead of detailed chemical kinetics mechanisms [27]. Detailed chemical kinetics would enable a more comprehensive description of gas species and intermediate reactions but are more computationally intensive. As such, molecular dynamics (MD) simulations with a reactive force field (ReaxFF) have significant potential to be applied to gain a more in-depth knowledge of pyrolysis breakdown of material and extract the key input data required for fire modelling.

ReaxFF proposed by van Duin et al. [28] is an empirical bond-order-based reactive forcefield capable of explicitly describing detailed bond breaking, bond formation, and subsequent complex chemical reactions within a molecular system. Owing to the consideration of bond disassociation and bond formation, ReaxFF is a computationally efficient approach to investigate detailed pyrolysis mechanisms of the thermal decomposition process. It can also provide formation pathways of different primary products and extract pyrolysis kinetics in thermal decomposition simulations. Therefore, the adoption of MD simulation would benefit the investigation of polymers’ thermal degradation at atomistic levels. For example, Chen et al. [29] characterised the pyrolysis process of three common engineered polymers (high-density polyethylene, poly (methyl methacrylate) and high impact polystyrene) through ReaxFF simulations, obtained detailed pyrolysis kinetics and char formation that was in good agreement with the experimental result. Varri and Paajanen [30] have carried out a ReaxFF based MD simulation to explore the effect of aluminium trihydroxide (ATH) on the thermal decomposition of polyethylene. The simulations replicated the endothermic decomposition of ATH into alumina and water. The simulations also revealed the chemical reaction between polyethylene and ATH, such as hydrogen abstraction, water production and enhanced charring. Rahmani et al. [31] examined both non-isothermal and isothermal decomposition of polyethylene oxide using reactive molecular dynamics simulation. The polyethylene oxide was loaded with different concentrations of pristine graphene and graphene oxide nanoplatelets. The result of the MD studies identified improvement in thermal stability by introducing pristine graphene to the polymers. Lan et al. [32] utilised MD simulations to investigate the atomistic behaviours of ammonium polyphosphate filled flame retarded polypropylene composites. The compatibility of flame retardants in the polymer matrix was optimised with different additives, which agrees with experimental data.

**Figure 1:**
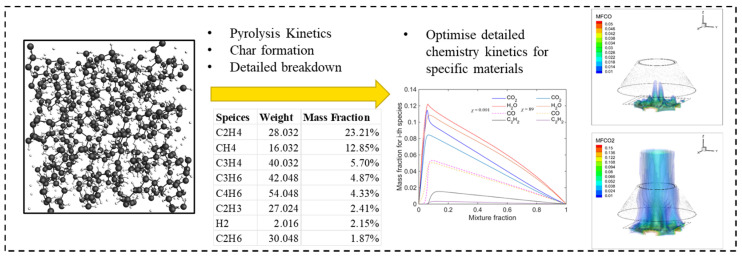
Multi-scale modelling framework incorporating molecular dynamics simulation with computational fluid dynamics.

All the reviewed works demonstrated MD simulations as a viable tool for investigating the pyrolysis chemistry of polymer systems and highlights the capability to identify the detailed decomposition process from solid to gas phases, which could further act as the precursors of combustible fuel gases in CFD combustion models. Although it is suggested that the turbulent transfer coefficients also plays an essential role, surpassing the molecular transfer coefficients in the numerical investigations [22,23,24,25]. MD simulations are able to identify the pyrolysis behaviour and flame retardancy mechanism at the molecular level. This study proposes a multi-scale modelling approach by applying the kinetics parameters and detailed pyrolysis gas volatiles (i.e., parent combustion fuel, key precursor species) extracted from MD to enable detailed chemistry modelling in CFD (see Figure 1). This methodology will deliver a more accurate prediction on polymer degradation, toxicity and smoke emission compared to current assumed CFD models. To investigate the validity of the proposed approach, numerical simulations will be performed on both polyethylene and flame retardant polyethylene. This work is expected to contribute towards future studies investigating the viability of coupling MD with CFD pyrolysis modelling and creating the framework for a fully coupled interactive fire model.

## 2. Results and Discussion

In order to model the pyrolysis process, the CFD model requires (i) the thermal properties of the material, (ii) the thermal degradation rate and (iii) gas-phase volatile releases involved during the pyrolytic process. Accordingly, instead of extracting these key data from a number of different fire experiments or empirical expressions for the estimation of these quantities, MD (ReaxFF) is employed. Specifically, MD simulations are performed to calculate (i) the thermal conductivity of the polymer system, (ii) the pyrolysis reaction kinetics and (iii) identify the detailed distribution of combustible volatiles of the polymer composites. The data were applied as inputs into a three-dimensional LES fire model comprising of (i) solid pyrolysis, (ii) gas-phase combustion, (iii) radiation heat exchange between fire source, walls, and gaseous products, (iv) soot formation, and (v) sub-grid scale (SGS) turbulence models. Finally, the simulation results are validated against experimental data from cone calorimetry.

### 2.1. Molecular Systems

**Figure 2:**
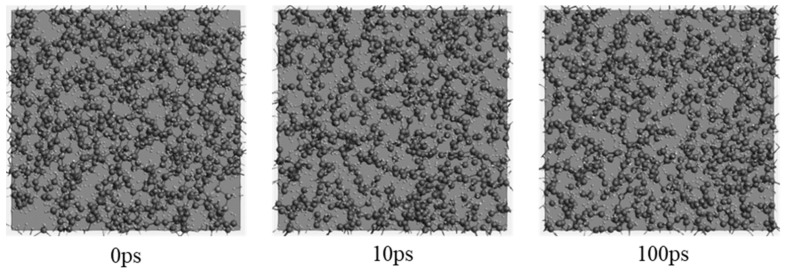
Snapshots of the evolving pyrolysis fragments at 3200 K for the polyethylene (PE) system.

**Figure 3:**
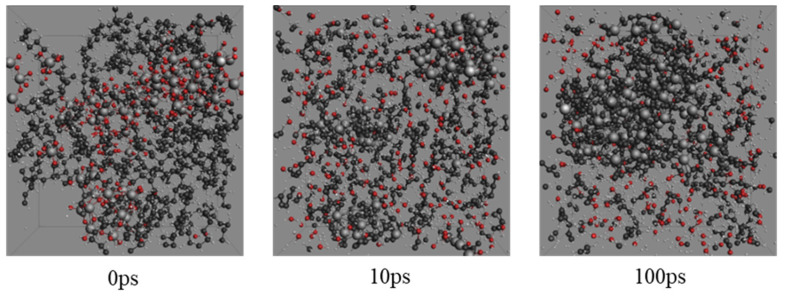
Snapshots of the evolving pyrolysis fragments at 3200 K for the polyethylene with 25 mass percentage aluminium trihydroxide (PE + 25 wt% ATH) system.

Figure 2 and Figure 3 reveal the snapshots of the atomic configurations of pure PE and PE filled with 25 mass percentage (wt%) ATH during the MD simulations at 3000 K. As can be seen from the snapshots, the detailed disassociation and formation of chemical bonds from the pyrolysis process can be observed at an atomic level over time. In the case of pure PE (Figure 2), the initial status of the simulation begins with the packed amorphous structure in the periodic domain. The bonding force between monomers begins disassociation when sufficient heat energy is applied to the system. Over an instantaneous time (i.e., 10 ps), it can be observed that the polymer structure initiates the breakdown process, where smaller molecular compounds are formed. With a longer duration of simulation time, there will be a further amount of minor chemical compounds forming. While for the ATH filled PE system (Figure 3), H_2_O molecules are formed owing to the existence of OH anions from the ATH. Except for the yields of water, it can also be observed that the breakdown of the PE grain is relatively slower than the pure PE system. The results highlight the capabilities of MD to analyse the detailed temporal distribution of the detailed pyrolysis breakdown gas products from thermal decomposition.

### 2.2. Pyrolysis Kinetic Analysis

**Figure 4:**
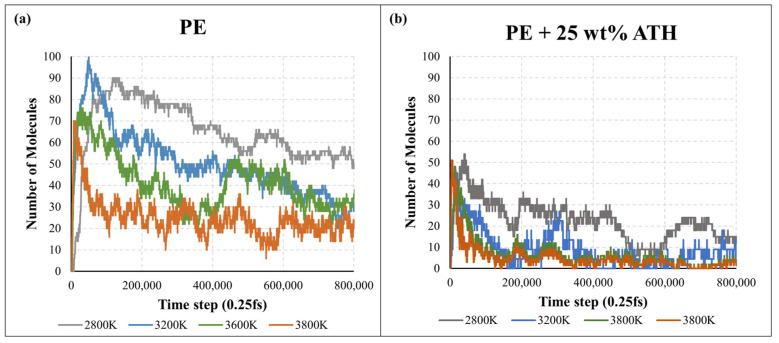
Numbers of C_2_H_4_ molecules for the (**a**) polyethylene (PE) and (**b**) polyethylene with 25% aluminium trihydroxide (PE + 25 wt% ATH) during the molecular dynamics’ simulation.

**Figure 5:**
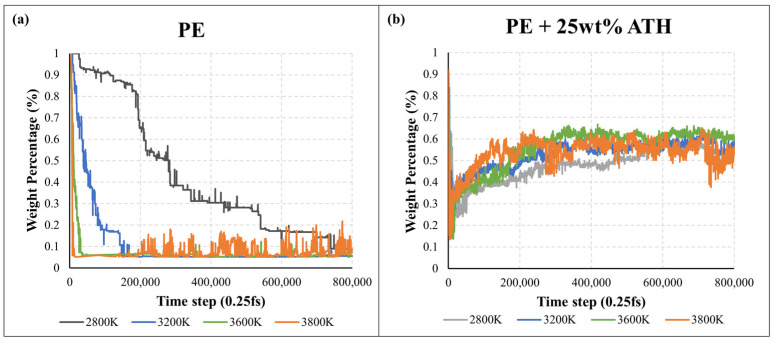
Weight percentage of C_40+_ for the (**a**) polyethylene (PE) and (**b**) polyethylene with 25% aluminium trihydroxide (PE + 25 wt% ATH) during the molecular dynamics simulation.

In the past few years, there are a few methods that have been investigated to approximate the thermal decomposition of polymers in MD. These include kinetics analysis on (i) the number of monomer molecules [33,34], and (ii) the approximation of mass loss by applying a threshold or cut-off based on carbon (C) number [35,36] or molecular weight [37,38,39]. This study determined the first-order pyrolysis kinetics by two different analysis approaches. The first approach (MD) analysed the molecular number of the backbone monomer (e.g., ethylene (C_2_H_4_)) at a temperature ranging from 2800 K–3800 K. While in the second approach (MD-CN), the kinetics were estimated by adopting a molecular weight cut-off filter at the temperature ranging from 2800 K–3800 K. Fletcher et al. suggested that the pyrolysis fragments can be classified as char species/residues (C_40+_), tar species (C_5–40_) and gas species (C_0–5_) based on the containing number of carbon atoms [40]. Figure 4 and Figure 5 demonstrate the time profile of the number of ethylene monomers and weight percentage of C_40+_ residues in the PE and PE + 25 wt% ATH simulations at different temperatures for the first 200 ps.

In Figure 4, a sharp increase in the number of ethylene monomers can be generally observed at the start, indicating the initial breakdown of the polymer into its monomer components. It is followed by a gradual decrease as the monomers break down into subsequent pyrolysis products. It can be clearly seen that the total numbers of ethylene in PE/ATH are less than PE, indicating the polymeric degradation rate of PE/ATH was slower. Figure 5 reveals the weight percentage of C_40+_ for the PE and PE/ATH during the molecular dynamics simulation. For pure PE (except 2800 K), the weight percentage of the pyrolysis fragment was rapidly degraded to 10% within the first 50 ps, indicating the violent decomposition of the polymers without any FR additives. While for PE/ATH (Figure 5b), the weight percentages of C_40+_ raised after the initial breakdown of PE during the first 50 ps, indicating the char formation led by the ATH additives.

As mentioned previously, the pyrolysis kinetics for solid decomposition reaction can be expressed in the form of Arrhenius expression:

(1) $$r=A\;exp\left(\frac{E_{a}}{RT}\right){\left(Y\right)}^{n}$$

where r is the reaction rate, $E_{a}$ is the activation energy, A is the pre-exponential, R is the universal gas constant, T is the reaction temperature, *Y* is the mass fraction of the solid material and *n* is an exponential factor for the mass fraction. The pyrolysis kinetic parameters $E_{a}$ and A were determined by analysing the correlation between the reaction rate $r_{i}$ and the corresponding pyrolysis temperature $T_{i}$, where *i* denotes the range of temperature. A linear relationship can be established between $\ln(r_{i})$ and $\left(1/T_{i}\right)$. The theoretical linear relationship between the two terms can be expressed as:

(2) $$\ln(r_{i})=\ln(A)-\frac{E_{a}}{R}\left(\frac{1}{T_{i}}\right)$$

**Figure 6:**
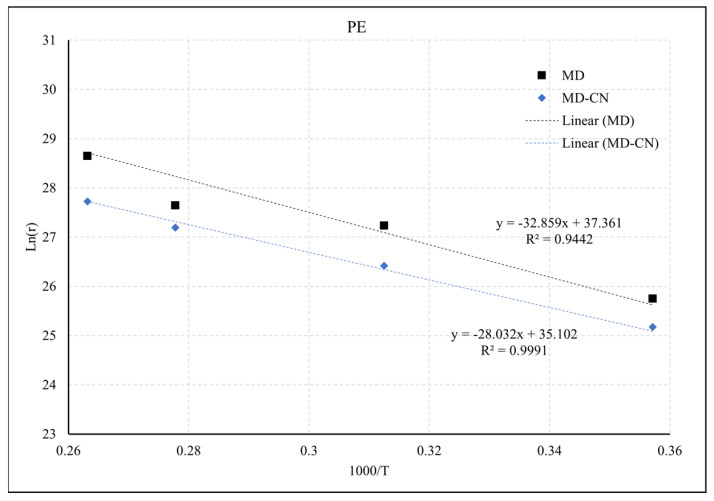
Fitted rate constant versus inverse temperature obtained from the MD simulations based on molecular number of C_2_H_4_ (MD) and cut-off filter by carbon number C_40+_ (MD-CN).

As illustrated in Figure 6, utilising Equation (2), the pyrolysis kinetic parameters $E_{a}$ and A can be extracted from the slope $\left(-E_{a}/R\right)$ and intercept (ln(A)) of the fitted line. The results are listed in Table 1. As can be seen, the results derived from MD were aligned with values obtained from TGA and also in the range of the values reported in Sinfronio et al. [41] of 227.33–269.04 for $E_{a}$ and 2.4 × 10^13^–1.8 × 10^16^ for A. Similarly, these approaches can also be used to extract the reactions for PE/ATH.

**Figure 7:**
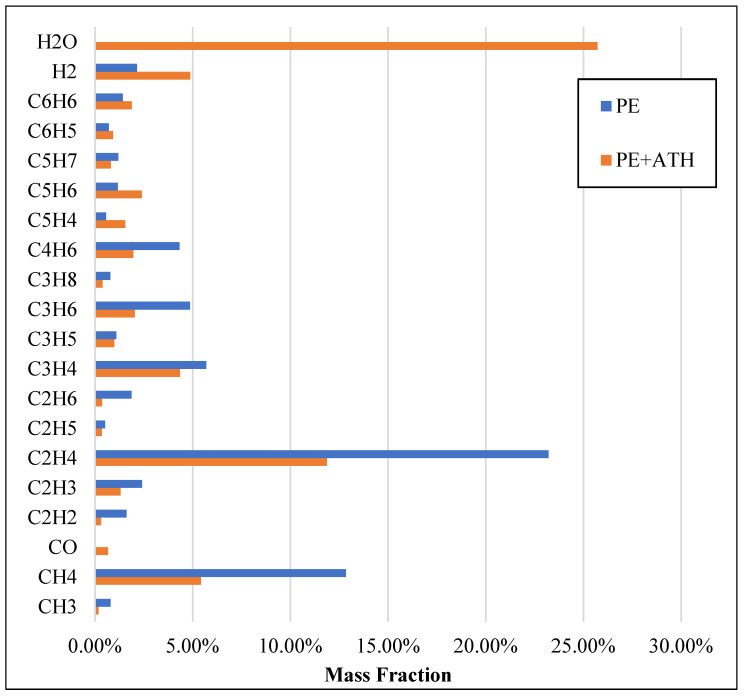
Detailed breakdown of the pyrolysis products for polyethylene (PE) and polyethylene with 25% aluminium trihydroxide (PE + ATH) arranged by mass fraction.

For the PE + 25% ATH case, it can be observed that the breakdown rate of the ethylene of the system was reduced due to the existence of ATH. As illustrated in Figure 7, the formation of H_2_O has significantly occurred in the PE + ATH simulations, which further consumed hydrogen atoms and reduced the formation of other alkane fuel species (e.g., methane CH_4_, ethylene C_2_H_4_ and propane C_3_H_6_) that requires hydrogen atoms. It can be noticed that the mass fraction of these alkane fuels in PE/ATH is significantly lower than in the pure PE simulations.

### 2.3. Incorporating MD into Detailed Chemistry Kinetics

**Figure 8:**
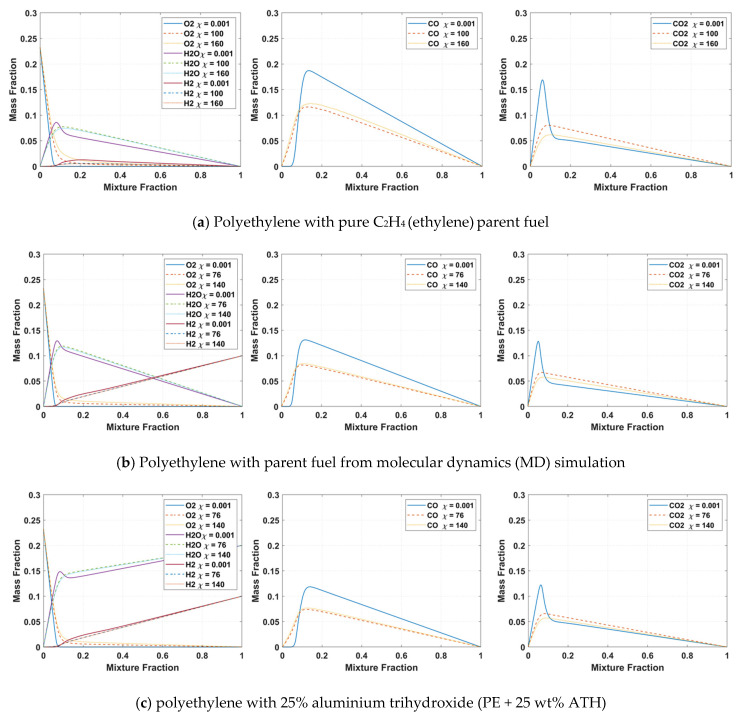
Flamelet profiles for selective major combustion species and carbon monoxide (CO) and carbon dioxide (CO_2_) at different scalar dissipation rates for (**a**) Polyethylene case with pure C_2_H_4_ (ethylene) parent fuel, (**b**) Polyethylene case with volatiles gas mixture from molecular dynamics simulation as the parent fuel and (**c**) polyethylene with 25% aluminium trihydroxide (PE + 25 wt% ATH) case with volatiles gas mixture from molecular dynamics simulation as the parent fuel.

The detailed chemical reaction mechanism GRI-Mech 3.0 [32] and CHEMKIN 19.2 [33] were used to generate the flamelet library for the combustion model. The initial 53 species flamelet library was reduced to 16 species by applying a mixture fraction cut-off to optimise the computational efficiency of the detailed chemistry model. The list of species includes H_2_, H, O, O_2_, OH, H_2_O, HO_2_, CH_3_, CH_4_, CH_2_O, CO, CO_2_, C_2_H_2_, C_2_H_4_, C_2_H_6_, C_3_H_6_, N_2_. The major selective combustion and toxic species at the maximum and minimum scalar dissipation rates for the three cases are displayed in Figure 8. From the flamelet profiles, it can be observed that the mass fraction of major combustion products, such as CO_2_ and H_2_O is at a maximum at around the stoichiometric where complete combustion occurs, passing the stoichiometric results in intermediate species, such as CO and as incomplete combustion occurs.

Figure 8a,b shows the flamelet profiles generated using 100% C_2_H_4_ as the parent fuel and the volatiles gas mixture characterised via MD simulation, respectively. Comparing both profiles, it can be seen that the flamelet profiles from MD simulation resulted in a noticeable decrease in both CO and CO_2_ and an increase in H_2_O and H_2_. More specifically, applying pure C_2_H_4_ as the parent fuel would result in little to no H_2_ formations, which is unrealistic since it is widely known that significant hydrogen is produced from the polymer pyrolysis process. Subsequently, many fire codes implement a prescribed H_2_ yield to overcome this issue. Another side point to note is that the scalar dissipation rate close to flame extinction is approximately 176 for pure C_2_H_4_ and decreases to approximately 150 when the MD gas mixture was applied. The scalar dissipation represents the level of flame straining/stretching caused by turbulence, leading to the non-uniformity of the mixture fraction from the chemical equilibrium. Focusing on Figure 8c, which shows the flamelets profiles for PE/ATH, it can be seen that there is a further decrease in CO and CO_2_ formations and is replaced with a significant increase in H_2_O formation. As previously identified from the MD simulation, ATH operates by a dehydration mechanism that releases water vapour (H_2_O), causing a dilution of combustible gas and oxygen concentration. This dilution effect from ATH is reflected in the combustion flamelet profiles, with reduced combustion resulting in less CO and CO_2_ formation.

### 2.4. Cone Calorimeter Simulation

**Figure 9:**
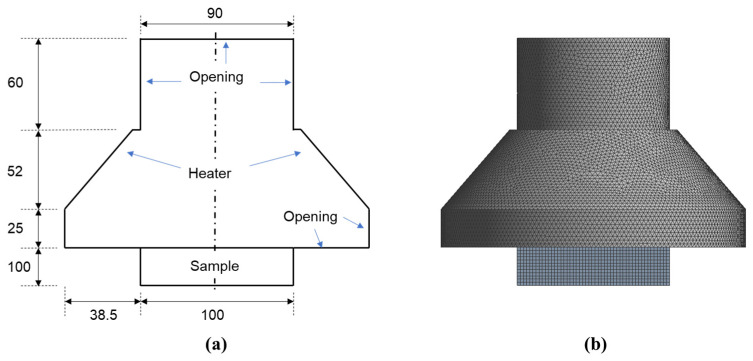
Diagram of (**a**) the cone calorimeter geometry with dimensions and (**b**) the meshed computational domain.

For validation and verification, numerical simulations have been performed based on the cone calorimeter experiment with computational geometry illustrated in Figure 9. The computational domain consists of the cone geometry, and a 60 mm long cylindrical extended region was applied from the cone outlet with a diameter of 90 mm.

**Figure 10:**
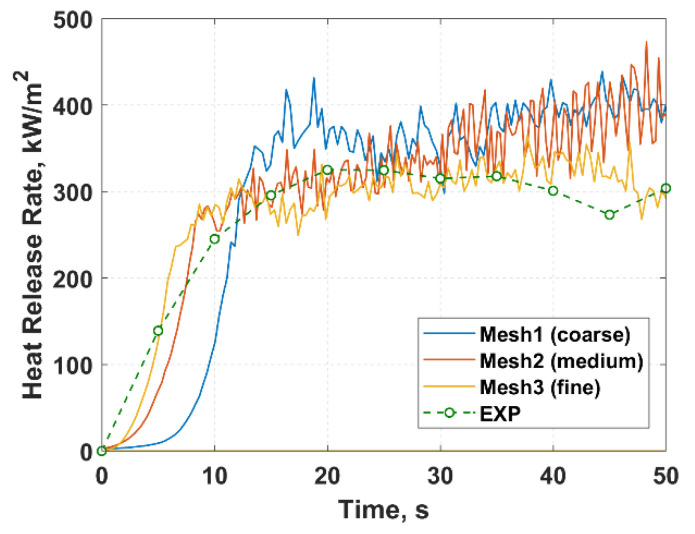
Mesh sensitivity comparison of the heat release rate profile for the three mesh systems.

A grid sensitivity analysis was performed on three mesh systems constructed based on the characteristic length analysis [42], detailed in Table 2. The heat release rate profile was used for comparison, as presented in Figure 10. The heat release rate for the first 50 s was used to analyse mesh convergence to reduce the computational time required for the test. The results show that the ignition time changed significantly from approximately 5.50 s for the coarse mesh to 2.37 s for the medium mesh (56.9% difference). Comparing the medium to fine mesh, the ignition time further converged from 2.37 s to 2.12 s with a difference of 6.3%. Furthermore, it can be observed that the mesh refinement resulted in a decrease in the overall heat release rate and converged to match more closely with the experimental data. Considering the convergence results, the 1.5 mm uniform mesh was adopted in this numerical simulation.

**Figure 11:**
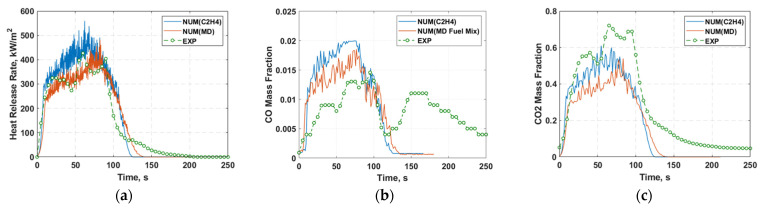
Comparison of numerical results against experimental (**a**) heat release rate (HRR), (**b**) carbon monoxide (CO) and (**c**) carbon dioxide (CO_2_) profile from cone calorimetry under a heat flux of 35 kW/m^2^ for (i) Polyethylene case with pure ethylene (C_2_H_4_) parent fuel, and (ii) Polyethylene case with volatiles gas mixture from molecular dynamics (MD) simulation as the parent fuel.

Figure 11a presents the numerically predicted heat release rate (HRR) profiles of the polyethylene cone simulations incorporating pure C_2_H_4_ (ethylene) and volatiles gas mixture from molecular dynamics simulation as the combustion chemical kinetics fuel precursors. The overall experimental trend of the heat release rate profiles was successfully captured in the numerical simulations. The single heat release rate peak predicted by the numerical model was in agreement with experimental data. Furthermore, considering the evaluated fuel gas volatile composition resulting from the MD pyrolysis breakdown delivers a more realistic HRR profile that closely matches the cone calorimeter experiment. In contrast, the assumption that pure C_2_H_4_ as the parent fuel yielded an overestimation of the HRR profile since the purity of the combustible volatile by right should not be 100% after the pyrolytic reactions, which this behaviour can be reflected in the MD model.

Figure 11b,c show the concentrations of the carbon monoxide (CO) and carbon dioxide (CO_2_) contents at the top cone outlet of the computational domain compared to experimental measurements. The results present the effects of changing the parent fuel based on molecular dynamics simulation on the formation of toxic by-products. It can be observed that by assuming the combustion fuel precursor to be purely C_2_H_4_, the amount of CO/CO_2_ concentrations generated is greater than that compared to the cases with the MD inputs.

**Figure 12:**
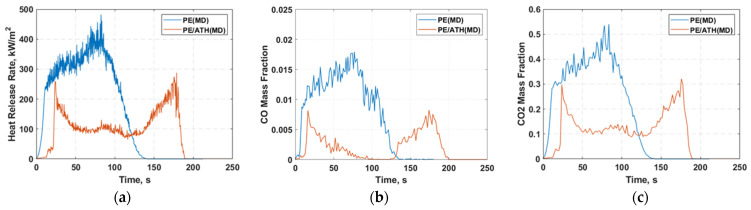
Comparison of numerical results against experimental (**a**) heat release rate (HRR), (**b**) Carbon Monoxide (CO) and (**c**) Carbon Monoxide (CO_2_) profile from cone calorimetry under a heat flux of 35 kW/m^2^ for (i) Polyethylene (PE) and (ii) polyethylene with aluminium trihydroxide (PE/ATH) case with volatiles gas mixture from molecular dynamics (MD) simulation as the parent fuel.

Figure 12 illustrates the numerically predicted heat release rate profiles of the simulations between PE and PE/ATH based on MD input. As shown in Figure 12a, the peak heat release rate was reduced by 34.2% compared to pure PE. It can be observed that the reduction in heat release rate led by the addition of ATH flame retardant is replicated in the cone simulations.

**Figure 13:**
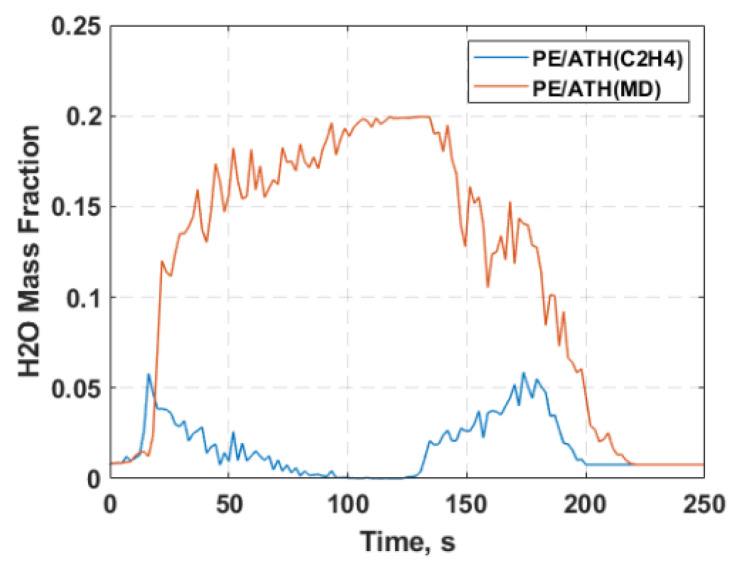
Numerical results for H_2_O mass fraction profile from cone calorimetry under a heat flux of 35 kW/m^2^ for polyethylene with aluminium trihydroxide (PE/ATH) case with (i) pure C_2_H_4_ (ethylene) and (ii) volatiles gas mixture from molecular dynamics (MD) simulation as the parent fuel.

The concentration of H_2_O species for the PE/ATH simulation applying C_2_H_4_ and MD inputs as the parent fuel is depicted in Figure 13. The generation of H_2_O species translates to water vapour formation, which is generally recognised as one of the major fire suppression mechanisms. As can be seen from the figure, the amount of H_2_O content significantly increases from PE/ATH when the MD inputs were applied compared to a pure reaction fuel. As mentioned previously, the intrinsic vaporisation properties of ATH when incorporated into PE promote a supplementary chemical pathway for rapid vaporisation, leading to increased moisture level after the pyrolytic process. Therefore, before exhibiting the combustion process, there was a considerable H_2_O content. In addition, the increased formation of H_2_O will also result in a reduction in combustible fuel gas volatile, thus further lowering the heat release rate during the burning process. The result demonstrates the application of the proposed MD framework to extract the flame retardant mechanisms to the CFD combustion model and provide a better representation of the gas decomposition process. It allows the CFD model to simulate the underlying flame retardant mechanism offered by ATH that suppresses the peak heat release rate.

## 3. Materials and Methods

The mathematical models and characterisation methods are presented in the following sections.

### 3.1. Molecular Dynamics

ReaxFF is an empirical bond-order-based reactive forcefield and can explicitly describe chemical reactions within complex systems. It is commonly applied to describe the general relationship between bond distance and bond order and between bond order and bond energy. This complex interaction leads to the proper dissociation of bonds to separated atoms. The molecular movement and inter-atomic interactions are governed by Newton’s second law:

(3) $$\sum F=\;ma=\;m\frac{dv}{dt}\;=\;m\frac{d^{2}x}{dt^{2}}$$

(4) F= −∇E

where *F* is the instantaneous force acting on a particle with mass *m*, acceleration *a*, *v* and *x* are the instantaneous particle velocity and position respectively, and *t* is the time. *E* the potential energy function and ∇ is the differential operator. MD is based on the general relationship between bond distance and bond order and the bond energy that leads to the dissociation of bonds to separate atoms. The energy function can be determined by:

(5) $$E_{system}=E_{bond}+E_{over}+E_{under}+E_{val}+E_{pen}+E_{tors}+E_{conj}+E_{vdWaals}+E_{Coulomb}$$

where all the *E* terms present the energy associated with the bond, over-and under-coordinated atom, valence angle term, penalty energy, torsion energy, conjugation effects to molecular energy, nonbonded van der Waals interaction and Coulomb interaction, respectively. The LAMMPS Molecular Dynamics Simulator [43] was applied to perform the numerical simulations. The reac/c package [44] was used to compute the ReaxFF potentials.

#### Molecular System Configuration and Simulation Details

**Figure 14:**
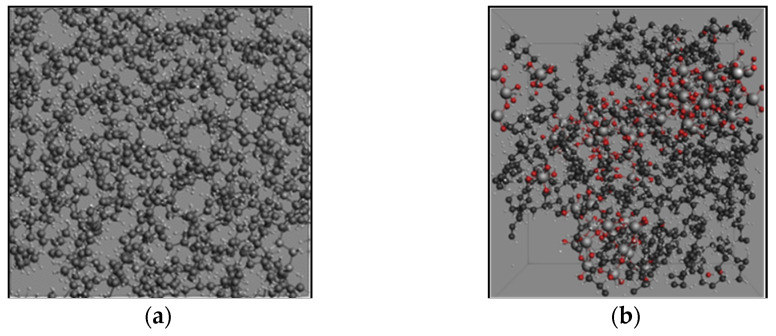
Amorphous structure of the (**a**) PE and (**b**) PE + ATH system.

In the current study, two materials were considered in the ReaxFF simulations, namely PE and aluminium trihydroxide (ATH). Owing to the existence of aluminium atoms, the reactive forcefield “AlCHO” proposed by Hong et al. was adopted [45]. Firstly, the molecular structure of PE was constructed by the compression of linear polymer chains, followed by an annealing process to form an amorphous structure, where the initial structures were slowly heated from 300 K to 600 K and rapidly quenched to 300 K. The subsequently annealed geometries were then relaxed using a conjugate gradient minimisation scheme. The initial model structure was created by PACKMOL [46] comprising of 4 linear PE chains (n = 50) in a 20 nm × 20 nm × 4 nm simulation box (200 carbon atoms in total), with a density of 0.93 g/cm^3^. Similar to the approach adopted by Varri and Paajanen [30], Gibbsite was introduced to represent the crystal structure of ATH, where two dioctahedral layers are related horizontally. The monoclinic unit cell comprised of two planes has 16 Al(OH)_3_ units. Five unit cells (80 Al(OH)_3_ units, 560 atoms) were then implemented through PACKMOL to achieve the proposed weight percentage in the current study (25 wt%). The final structures of the PE and PE/ATH are shown in Figure 14. For the thermal decomposition simulations involving pure PE and PE/ATH composites, the temperature and pressure of the system were regulated using the Nose-Hoover thermostat and barostat with a damping coefficient of 100 fs [47]. Various MD-ReaxFF studies on polymer degradation have suggested high artificial temperatures to promote sufficient atomic motion and molecular collision, with reasonable computational cost [29,36,48,49,50,51]. Hence, the simulations were carried out for 200 ps in a range of artificial temperature from 2800 K to 3800 K for PE and PE/ATH composites pyrolysis analysis.

### 3.2. Computational Fluid Dynamics

The fire model was developed using ANSYS Fluent version 19.2, extending a three-dimensional porous media pyrolysis model from previous work [4]. The solid pyrolysis model was developed with user-defined functions (UDF) which describe the solid thermal degradation process and porous properties of the sample. UDF allows customization for boundary conditions [52], material properties [53,54], source terms [4] and model parameters [55,56]. The Wall-Adapting Local Eddy-viscosity was employed to resolve the subgrid-scale eddies, and the Moss-Brookes semi-empirical soot model was implemented to handle the soot concentration within the cone computational domain [57]. The complex fluid motion and heat transfer of a turbulent reacting non-premixed diffusion flame is governed by the equations of mass, momentum and energy. Conservation of scalar properties, such as gas chemical species is governed by a transport equation. For practical simulation of a weak buoyancy-driven flame, several assumptions are made including (i) the low Mach number flow equations are considered, (ii) the thermophysical properties are constant and (iii) the ratio between mass and thermal diffusivity (Lewis number) is unity [58,59]. Accordingly, the following form of the governing equations are utilised in this simulation study:

**Mass conservation equation:**

(6) $$\frac{\partial \rho }{\partial t}+\nabla \cdot \left(\rho u\right)=S_{m}\;$$

**Momentum conservation equation:**

(7) $$\frac{\partial \bar{\rho }\tilde{u_{i}}}{\partial t}+\frac{\partial }{\partial x_{i}}\left(\bar{\rho }\tilde{u_{i}}\tilde{u_{j}}\right)=-\frac{\partial \bar{p}}{\partial x_{i}}-\frac{\partial {\bar{\tau }}_{ij}}{\partial x_{j}}-\frac{\partial \tau_{ij}^{sgs}}{\partial x_{j}}+\bar{\rho }g+\bar{{\dot{m}}_{b}^{\prime \prime \prime }\tilde{u_{b,i}}}\;$$

The term ${\dot{m}}_{b}^{\prime \prime \prime }u_{b,i}$ represents the bulk source term and $\tau_{ij}^{sgs}$ are the subgrid-scale stresses.

**Energy conservation equation:**

(8) $$\frac{\partial }{\partial t}\left(\rho h_{s}\right)+\nabla \cdot \left(\rho h_{s}u\right)=\frac{D\bar{p}}{Dt}+{\dot{Q}}_{c}^{\prime \prime }-{\dot{Q}}^{\prime }\;$$

where ${\dot{Q}}_{c}^{\prime \prime }$ represents the heat release rate per unit volume from a combustion reaction and ${\dot{Q}}^{\prime \prime }$ denotes the conductive, diffusive, and radiative heat fluxes:

(9) $${\dot{Q}}^{\prime \prime }=-k\nabla T-\underset{\alpha }{\sum }h_{s,\alpha }\rho D_{\alpha }\nabla Z_{\alpha }+{\dot{Q}}_{r}^{\prime \prime }\;$$

where k is the thermal conductivity and $D_{\alpha }$ is the diffusivity of species α.

#### 3.2.1. Pyrolysis Model

For the pyrolysis model, it is assumed that: (i) the release of fuel is instantaneous, (ii) there are no porosity and moisture effects, and (iii) the fuel is injected at the surface of the pyrolysing solid. The pyrolysis process is driven by the solid fuel temperature $T_{s}$.computed by the one-dimensional heat conduction equation at the direction to the depth of the solid fuel:

(10) $$\rho_{s}c_{s}\frac{\partial T_{s}}{\partial t}=\frac{\partial }{\partial x}\left[k_{s}\frac{\partial T_{s}}{\partial x}\right]+{\dot{Q}}_{p}^{\prime \prime }+{\dot{Q}}_{r}^{\prime \prime }\;$$

The source terms ${\dot{Q}}_{p}^{\prime \prime }$ and ${\dot{Q}}_{r}^{\prime \prime }$ refers to the net heat gain due to chemical reactions during pyrolysis and radiation absorption, respectively. The pyrolysis source term can be determined by the equation:

(11) $${\dot{Q}}_{p}^{\prime \prime }=-\rho_{s}\overset{N_{R}}{\underset{i=1}{\sum }}r_{i}{∆H}_{r,i}$$

where $H_{r,i}$ is the heat of reaction and $r_{i}$ is the thermal degradation rate computed in the form of the Arrhenius expression:

(12) $$r_{i}=c_{i}{\left(\frac{\rho_{s}}{\rho_{s0}}\right)}^{n_{i}}A_{i}exp\left(\frac{{-E}_{a,i}}{{RT}_{S}}\right)$$

As thermal degradation can consist of multiple parallel reaction mechanisms, the *i* subscript denotes the individual reaction components with the corresponding mass fraction $c_{i}$ and pyrolysis kinetic parameters $E_{a,i}$ and $A_{i}$. The total thermal degradation rate is determined by the sum of all reaction components and the amount of fuel releases ${\dot{m}}_{fuel}$ released at the material surface is provided as:

(13) $${\dot{m}}_{fuel}=\rho_{s}V\overset{N_{R}}{\underset{i=1}{\sum }}r_{i}\;$$

where V is the unit cell volume. It is assumed that the mass loss is fully converted into the parent fuel of the combustion model.

#### 3.2.2. Turbulence

The Wall-Adapting Local Eddy-viscosity (WALE) model developed Nicoud and Ducros [60], is based on the Smagorinsky [61] LES framework but is more effective at near-wall conditions and wall-bounded flows. The turbulent viscosity $\mu_{T}$ is given by:

(14) $$\mu_{T}=\rho L_{s}^{2}\frac{{\left(S_{ij}^{d}S_{ij}^{d}\right)}^{\frac{3}{2}}}{{\left(\bar{S_{ij}S_{ij}}\right)}^{\frac{5}{2}}+{\left(S_{ij}^{d}S_{ij}^{d}\right)}^{\frac{5}{4}}}$$

The mixing length for sub-grid scales $L_{s}$ and rate-of-strain tensor $S_{ij}^{d}$ are determined in the following equations:

(15) $$L_{s}=\min\left(\kappa d,C_{w}\Delta \right)$$

(16) $$S_{ij}^{d}=\frac{1}{2}\left({\tilde{g}}_{ij}^{2}+{\tilde{g}}_{ji}^{2}\right)-\frac{1}{3}\delta_{ij},\;{\tilde{g}}_{ij}^{2}=\frac{\partial {\tilde{u}}_{i}}{\partial x_{j}}$$

$C_{w}$ is the WALE model constant, prescribed as 0.5 validated previously for various fire simulation studies [62,63]. WALE detects the turbulent structure by the combination of strain and rotations rate so that the turbulent viscosity term will be null naturally at the wall boundary without the inclusion of a damping function.

#### 3.2.3. Detailed Chemistry Combustion

The strained laminar flamelet model for non-premixed combustion has been adopted. It assumes the fuel burns instantly when mixed with the oxidiser. The combustion chemical source term which appears in the energy equation is calculated based on a fast-chemistry mixture fraction model. In this model, the detailed chemical kinetics of the oxidation process of the parent fuel was considered using the strained laminar flamelet approach. The temperature and chemical species generation (denoted by *m*) is determined by the multiple flamelets (*M*) as a function of mixture fraction f, fluctuation of mixture fraction $f^{\prime }$, and scalar dissipation term χ:

(17) $$m=\iint M(f,\;\chi )\;P\left(f,\chi ,f^{\prime }\right)\;dfd\chi$$

where P is the corresponding beta probability density function (PDF). The scalar dissipation χ is introduced to depict the level of flame straining/stretching leading to the non-uniformity of mixture fraction from the chemical equilibrium. The scalar dissipation is given by:

(18) $$\chi =C_{x}\frac{\left(\mu +\mu_{T}\right)}{\rho \sigma_{T}}{\left|\nabla f\right|}^{2}$$

The amount of heat generation via combustion is thus determined by the summation of species mass fraction multiplying its heat of formation.

(19) $${\dot{Q}}_{c}^{\prime \prime }=\frac{1}{2}\rho \chi \overset{N}{\underset{i=1}{\sum }}\left[\overset{1}{\underset{0}{\int }}\left(h_{s_{i}}\frac{\partial^{2}M_{i}}{\partial \zeta^{2}}P\left(\zeta ,\zeta^{\prime },\chi \right)\right)d\zeta \right]$$

where $h_{s_{i}}$ depicts the heat of formation of the corresponding *i*-th species. The detailed chemical reaction mechanism GRI-Mech 3.0 [64] and CHEMKIN 19.2 [65] was used to generate the flamelet library for the combustion model. GRI-Mech 3.0 is very well-validated and shown to produce accurate and reliable results for alkane fuels [11,55,58].

#### 3.2.4. Soot Formation

The Moss-Brookes semi-empirical soot model [57] was implemented where acetylene is considered as the soot precursor. Numerical studies have shown that the Moss-Brookes soot model combined with detailed kinetics results in significant improvements in the prediction of soot volume fraction [66,67]. This model is a two-equation semi-empirical soot model where acetylene is considered as the soot precursor and solves transport equations for normalised radical nuclei concentration $b_{nuc}^{*}$ and soot mass fraction $Y_{soot}$:

(20) $$\frac{\partial }{\partial t}\left(\rho Y_{soot}\right)+\nabla \cdot \left(\rho Y_{soot}\tilde{u}\right)=\nabla \cdot \left(\frac{\mu_{t}}{\sigma_{soot}}Y_{soot}\right)+\frac{dM_{soot}}{dt}$$

(21) $$\frac{\partial }{\partial t}\left(\rho b_{nuc}^{*}\right)+\nabla \cdot \left(\rho b_{nuc}^{*}\tilde{u}\right)=\nabla \cdot \left(\frac{\mu_{t}}{b_{nuc}^{*}}b_{nuc}^{*}\right)+\frac{1}{10^{15}}\;\frac{dN_{soot}}{dt}$$

where $M_{soot}$ is the soot mass concentration, $N_{s}$ denotes soot particle number density.

#### 3.2.5. Radiation

The radiative heat transfer was modelled using the filtered radiative transfer equations (FRTE) for non-scattering gray gas solved by the discrete ordinates method (DOM) with the S4 quadrature scheme [68]. The discrete radiation source term ${\dot{Q}}_{r}^{\prime \prime }$ that appears in the energy equation is determined as:

(22) $${\dot{Q}}_{r}^{\prime \prime }=-4{\bar{k}}_{a}E_{b}+\overset{24}{\underset{j=1}{\sum }}w_{j}{\bar{k}}_{a}{\bar{I}}_{j}$$

where the blackbody radiation is represented by $E_{b}=\sigma T^{4}$, σ is the Stefan-Boltzmann constant and ${\bar{I}}_{j}$ is the radiation intensities that span over the solid angles range of 4π around a point in space. The filtered gas absorption coefficient ${\bar{k}}_{a}$ was determined as a summation of the gas absorption coefficient approximated using the Weighted Sum of Gray Gases Model [69] and soot [70].

### 3.3. Experiment

#### 3.3.1. Thermogravimetry

Thermogravimetry is a proven method to study the thermal decomposition of a material. The thermal gravimetric (TGA) analysis was carried out on a Netzsch TG 209 F3 Tarsus thermoanalyser instrument (Netzsch, Selb, Germany) from room temperature (21 °C) to 1000 °C in a nitrogen atmosphere under three different heating rates, namely 5, 10 and 20 K/min. The specimens were approximately 10 mg in mass, and each test was performed twice to ensure consistency of the data.

#### 3.3.2. Cone Calorimetry

The flammability (i.e., heat release) of the material samples was examined via Cone Calorimetry according to ISO 5660 standards to study the flaming behaviour (i.e., ignition time, heat release rate and burn duration) and their corresponding smoke, CO_2_ and CO production. The tests were performed on an iCone Classic Calorimeter (Fire Testing Technology, East Grinstead, UK). All samples were cut to 100 mm × 100 mm, then wrapped in aluminium foil with the upper surface exposed. The distance between the cone heater and test surface was set as 25 cm. The measurements were carried out by mounting the sample holder in the horizontal position under atmospheric conditions with a nominal exhaust fan airflow rate of 0.0026 m^3^/s for all experiments. Three samples of each material were examined under 35 kW/m^2^ incident heat flux.

## 4. Conclusions

The fundamental thermal degradation process from solid fuel to gas volatiles is still one of the major challenges in the field of pyrolysis modelling. A multi-scale modelling approach was proposed to address this significant knowledge gap by applying the kinetics parameters and detailed pyrolysis gas volatiles (i.e., parent combustion fuel, key precursor species) extracted from Molecular Dynamics (MD) to enable a more realistic detailed chemistry combustion in Computational Fluid Dynamics (CFD) fire model. This multi-scale modelling is a potential technique for the simulation of combustible polymer materials, as it allows to describe them without the need of performing costly experiments. In this study, the key properties associated with the burning behaviour of pure polyethylene (PE) and polyethylene with aluminium trihydroxide (PE/ATH) were analysed by molecular dynamics simulations. The microscopic pyrolysis behaviours of the polymers investigated by molecular dynamics were in agreement with the TGA data. The pyrolysis reaction kinetics were successfully calculated by analysing the breakdown rate of the underlying monomer structures at different temperatures. Focusing on the PE with ATH, the MD simulations were able to show the pyrolysis process as the ATH component leads to the increased formation of H_2_O molecules. Subsequently, it leads to a reduced amount of combustible fuel gas volatile, thus reducing the combustion by-products and heat release rate.

From the MD simulations, the material kinetics parameters were directly applied as inputs into a CFD pyrolysis model to simulate cone calorimeter experiments. The model incorporates strained laminar combustion modelling with detailed chemical kinetics, soot formation, subgrid scale turbulence, and radiation models. Furthermore, the detailed pyrolysis gas volatiles from MD was applied to generate the flamelet library for the combustion model. The overall trend of the heat release rate profiles was successfully captured in the numerical simulation. The carbon dioxide and carbon monoxide comparisons were also aligned with experimental results. Furthermore, considering the evaluated fuel gas volatile composition resulting from the molecular dynamics pyrolysis breakdown delivers a more realistic heat release rate profile that closely matches the cone calorimeter experiment than using a single parent fuel.

To summarise, the utilisation of the MD model delivers an in-depth understanding of the pyrolysis and chemical decomposition of molecules of the polymer composites. This allows us to realise the composition of fuel gas volatiles and incorporate them into CFD simulations models with a detailed chemistry combustion sub-module to interpret the data. The underlying flame retardant mechanisms can be described from a fundamental chemistry standpoint through this proposed framework. For instance, the rapid vaporisation effect offered by ATH was studied and its influence on the heat release rate reductions and chemical by-products formations were identified.

## Figures and Tables

**Table 1:** Pyrolysis kinetic parameters extracted from MD compared with experiments.

Polymer Type		*E_a_* (kJ/mol)	*A* (1/s)
PE	MD	273.25	1.798 × 10^16^
	MD-CN	233.06	1.76 × 10^13^
	TGA Experiment	266.74	1.52 × 10^16^
PE + ATH	MD	325.43	5.0291 × 10^11^
	MD-CN	285.41	5.03 × 10^13^

**Table 2:** Details of the mesh systems used in the mesh sensitivity analysis.

Mesh	Mesh Size (mm)	Total Number of Cells
Mesh 1 (coarse)	5	676,875
Mesh 2 (medium)	2	1,353,750
Mesh 3 (fine)	1.5	3,210,000

## Data Availability

The data presented in this study are not available from the authors.

## Nomenclature

A	Exponential factor
a	Acceleration
$b_{nuc}^{*}$	Normalised radical nuclei concentration
c_i_	Mass fraction
$C_{w}$	WALE model constant
D	Diffusivity
$E_{a}$	Activation energy
*E_system_*	System energy
*E_bond_*	Bond energy
*E_over_*	Over-coordinated atom energy
*E_under_*	Under-coordinated atom energy
*E_val_*	Valence angle term
*E_pen_*	Penalty energy
*E_tors_*	Torsion energy
*E_conj_*	Conjugation effects to molecular energy
*E_vdWaals_*	Nonbonded van der Waals interaction
*E_Coulomb_*	Coulomb interaction
F	Instantaneous force
∇f	Mixture fraction variance
g	Gravity
H	Enthalpy
$H_{R}$	Heat of reaction
$h_{s_{i}}$	Heat of formation
${\bar{I}}_{j}$	Radiation intensities
K	Thermal conductivity
${\bar{k}}_{a}$	Summation of the gas absorption coefficient
$L_{s}$	Mixing length for sub-grid scales
$M_{soot}$	Soot mass concentration
m	Mass
${\dot{m}}_{fuel}$	Fuel released at material surface
$N_{soot}$	Number density of soot particles
n_i_	Reaction order of the reaction
P	Pressure
Q	Heat released by the fluid combustion
${\dot{Q}}_{p}^{\prime \prime }$	Net heat gain due to chemical reactions during pyrolysis
${\dot{Q}}_{r}^{\prime \prime }$	Net heat gain due to chemical reactions via radiation absorption
$q_{r}$	Flux equation of thermal radiation
*R*	Gas constant
$R_{i}$	Pyrolysis reaction rates
$S_{ij}^{d}$	Rate-of-strain tensor
*T*	Absolute temperature
*Ts*	Solid fuel temperature
t	time
u	velocity
χ	Scalar dissipation
x	Instantaneous particle position
Y*_soot_*	Mass fraction of soot
Z	Elemental mass fraction
ρ	Density
$\tau_{ij}^{sgs}$	Subgrid-scale stresses
$\mu_{t}$	Turbulent viscosity
$\kappa_{d}$	Von Kármán constant

## Abbreviations

ATH	Aluminium (tri)hydroxide
C	Carbon
CFD	Computational Fluid Dynamics
CO	Carbon Monoxide
CO_2_	Carbon Dioxide
C_2_H_4_	Ethylene
FDS	Fire Dynamic Simulator
HRR	Heat Release Rate
H_2_O	Water
LES	Large Eddy Simulation
MD	Molecular Dynamics
MD-CN	Moleculary Dynamics Carbon Number Approach
PE	Polyethylene
ReaxFF	Reactive Forcefield
TGA	Thermogravimetric Analysis
WALE	Wall Adapting Local Eddy-viscosity
wt%	weight percentage
